# Refinement of diagnostic criteria for pediatric-type diffuse high-grade glioma, *IDH*- and H3-wildtype, *MYCN-*subtype including histopathology, *TP53*, *MYCN* and *ID2* status

**DOI:** 10.1186/s40478-023-01667-x

**Published:** 2023-10-24

**Authors:** Arnault Tauziède-Espariat, Emmanuelle Uro-Coste, Yvan Nicaise, Philipp Sievers, Andreas von Deimling, Felix Sahm, Oumaima Aboubakr, Alice Métais, Fabrice Chrétien, Pascale Varlet

**Affiliations:** 1 Department of Neuropathology, GHU-Paris Psychiatrie et Neurosciences, Hôpital Sainte Anne, 1, rue Cabanis, Paris, F-75014 France; 2 Department of Pathology, Toulouse University Hospital, Toulouse, France; 3 INSERM U1037, Cancer Research Center of Toulouse (CRCT), Toulouse, France; 4 Université Paul Sabatier, Toulouse III, Toulouse, France; 5 Department of Neuropathology, Institute of Pathology, University Hospital Heidelberg, Heidelberg, Germany; 6 Clinical Cooperation Unit Neuropathology, German Consortium for Translational Cancer Research (DKTK), German Cancer Research Center DKFZ), Heidelberg, Germany; 7 Institute of Psychiatry and Neuroscience of Paris (IPNP), Université Paris Cité, INSERM U1266, Ima- Brain team, Paris, 75014 France

Diffuse pediatric-type high-grade glioma (pHGG), *IDH*- and H3-wildtype are currently subdivided into three subgroups: pHGG-RTK (Receptor Tyrosine Kinase) 1, pHGG-RTK2, and pHGG-MYCN [2]. Each subgroup is known to present recurrent gene amplifications such as *PDGFRA, EGFR* and *MYCN*, respectively for RTK1, RTK2, and MYCN [2] but these amplifications are not specific to these subgroups. Although recurrent histopathological (such as nodules composed of large cells with prominent nucleoli and expression of glial and neuronal markers) [4, 5], and genetic (*TP53* mutations, particularly in a context of Li-Fraumeni syndrome) features [1] have been identified in pHGG-MYCN, the diagnosis of this subgroup is currently confirmed only by DNA-methylation analysis. Because a subset of pHGG-MYCN have been found to harbor an amplification of the *ID2* gene with (12/36, 33%) or without (1/36, 3%) a concomitant *MYCN* amplification (*MYCN+/ID2+*) [2], we have formulated a FISH (Fluorescence in situ hybridization) technique that targets both loci. Taking a monocentric series of 29 pHGG, *IDH*- and H3-wildtype, we studied the status of *MYCN* and *ID2* for each tumor, and correlated the data with histopathological and genetic features (including *TP53* status, somatic and germline), and DNA-methylation profiling.

The results are detailed in Supplementary Fig. 1 and Supplementary Table 1. The integrative histopathological, genetic and epigenetic analyses, including t-Distributed Stochastic Neighbor Embedding analysis (t-SNE) (Supplementary Fig. 2) segregated tumors into: pHGG-RTK1 (n = 5), pHGG-RTK2 (n = 11), and pHGG-MYCN (n = 13). All DNA-methylation proven pHGG-MYCN, except one (#11), harbored an amplification of *MYCN*, and five of them an *ID2* amplification (Fig. 1). A *MYCN* amplification was also observed in 31% (5/16) of pHGG-non MYCN (four RTK2 and one RTK1). However, none of them had an *ID2* amplification. Ten/13 pHGG-MYCN presented the histopathological features previously reported for this subgroup, whereas the three remaining cases showed features typically associated with diffuse astrocytic gliomas. Somatic mutations of *TP53* were present in 12/13 pHGG-MYCN, and four of them (with available data) harbored a germline mutation (Fig. 1). The outsider case (#11) presented the histopathological features of pHGG-MYCN, a *TP53* somatic mutation and a *MYC* amplification (Supplementary Fig. 3).

**Fig. 1:**
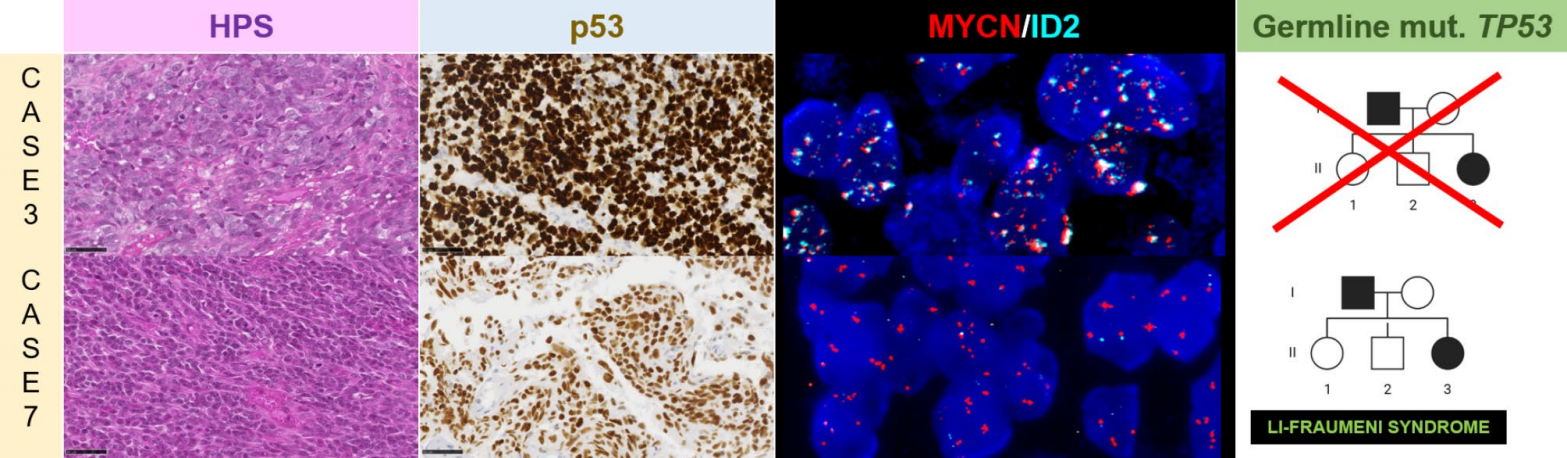
Histopathological and molecular features of pediatric high-grade gliomas, MYCN subgroup. The case #3 presented classical features of HGG-MYCN such as a dense proliferation composed of large cells with prominent nucleoli (HPS, magnification x400) with overexpression of p53 (magnification x400). The FISH analysis evidenced an amplification of both *MYCN* (orange signals) and *ID2* (blue signals) loci (magnification x800). There was no Li-Fraumeni syndrome in this case. The case #7 presented classical features of HGG-MYCN such as a dense proliferation composed of large cells with prominent nucleoli and numerous mitoses (HPS, magnification x400) with overexpression of p53 (magnification x400). The FISH analysis evidenced an amplification of *MYCN* locus without amplification of *ID2* gene (magnification x800). There was a context of Li-Fraumeni syndrome. FISH: Fluorescence in situ hybridization; HGG: high-grade glioma; HPS: Hematoxylin Phloxin Saffron; mut.: mutation. Black scale bars represent 50 μm

**Fig. 2:**
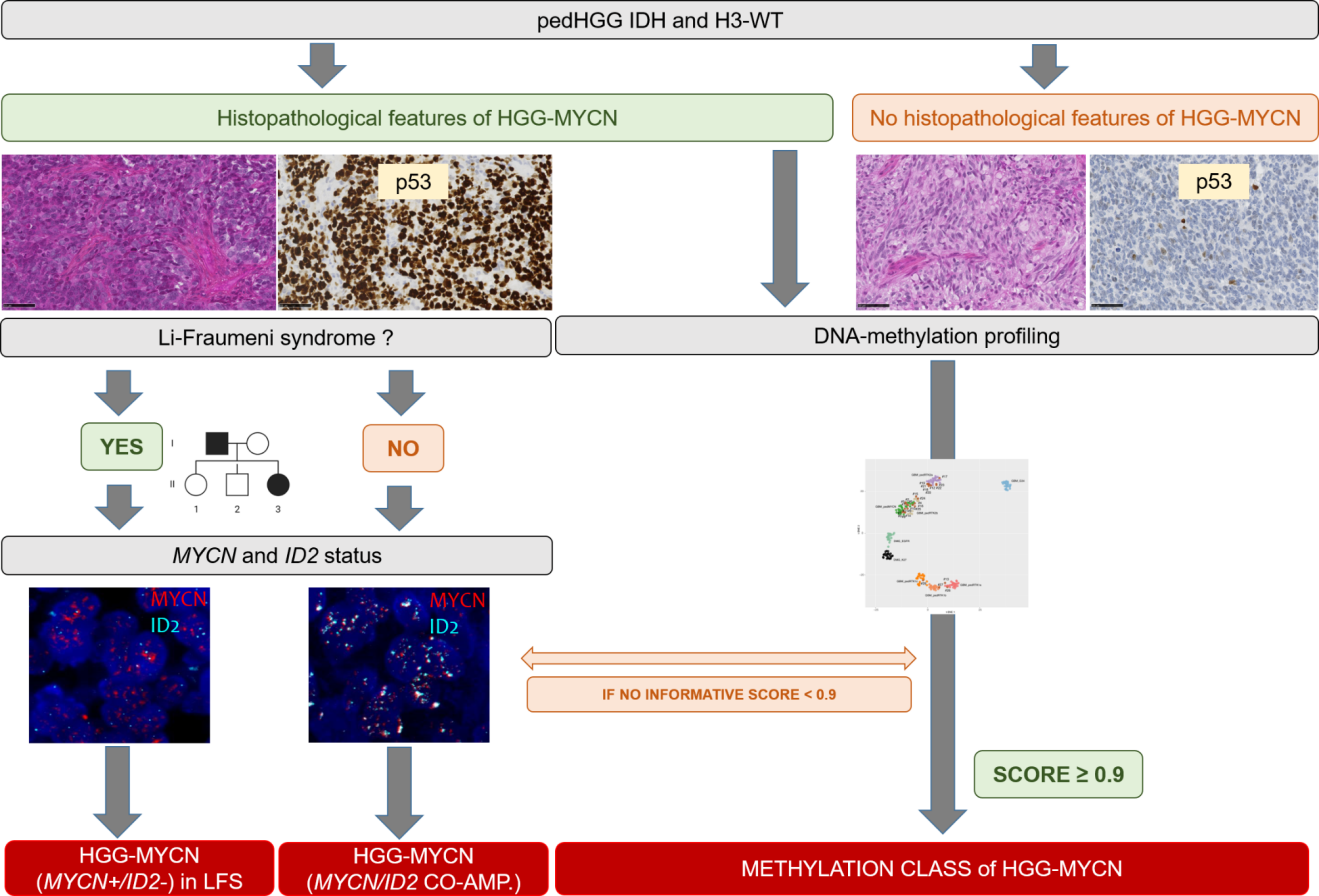
Diagnostic approach for pediatric high-grade glioma, with *MYCN*-amplification. Co-amp.: co-amplification; HGG: high-grade glioma; LFS: Li-Fraumeni syndrome; ped: pediatric; Wt: wildtype. Black scale bars represent 50 μm

As previously reported [2], a subset of pHGG-MYCN presented a co-amplification of *MYCN* and *ID2* (*MYCN+/ID2+*), which was specific for this subgroup of pHGG, and another subset of pHGG harbored an amplification of *MYCN* without *ID2* amplification (*MYCN+/ID2-*). The current series showed that all pHGG-MYCN cases, except one, harbored a somatic *TP53* mutation and a subset of them presented a germline mutation for *TP53*, as previously reported [1]. Interestingly, our results seem to show that pHGG-MYCN *MYCN+/ID2 +* are not associated with *TP53* germline mutations, whereas Li-Fraumeni syndrome is present in the subgroup of *MYCN+/ID2-*. Moreover, the current series described for the first time a pHGG-MYCN without amplifications of *MYCN* and *ID2* but harboring a *MYC* amplification. This example may illustrate the phenomenon previously described where a spinal ependymoma classified as “MYCN amplified” by DNA-methylation profiling but harbored a *MYC* amplification [3]. DNA-methylation analysis is still only limited to a small number of centres worldwide or may not be contributive (8/29 cases from this cohort presented a low calibrated score for a methylation class), so alternative methods for routine practice need to be validated. Therefore, histopathology (including p53 overexpression correlated to *TP53* mutation), and FISH *MYCN/ID2* may help diagnose pHGG-MYCN, using a simple algorithm approach (Fig. 2).

While a subset of pHGG-MYCN is found in the tumoral spectrum of Li-Fraumeni syndrome, for exclusion purposes, the co-amplication *MYCN/ID2* seems not to be associated with this genetic predisposition. The association of histopathological and genetic features may potentially represent alternative diagnostic criteria for pHGG-MYCN, particularly if DNA-methylation profiling is not available or not conclusive. FISH analyses of *MYCN/ID2* genes may constitute an interesting diagnostic tool for routine neuropathological practice.

### Electronic supplementary material

Below is the link to the electronic supplementary material.

**Supplementary Material 1: Figure S1. Clinical, histopathological, genetic and epigenetic characteristics of the cohort.**

**Supplementary Material 2: Figure S2. t-distributed stochastic neighbor embedding (t-SNE) analysis of DNA methylation profiles of the investigated tumors alongside selected reference samples.** Reference DNA methylation classes: diffuse midline glioma H3 K27M mutant (DMG_K27); diffuse midline glioma EGFR-altered (DMG_EGFR); diffuse high-grade glioma, H3.3 G34 mutant (GBM_ G34); pediatric glioblastoma, IDH wildtype, subclass MYCN (GBM_pedMYCN); pediatric glioblastoma, IDH wildtype, subclass RTK1a (GBM_pedRTK1a); pediatric glioblastoma, IDH wildtype, subclass RTK1b (GBM_pedRTK1b); pediatric glioblastoma, IDH wildtype, subclass RTK1c (GBM_pedRTK1c); pediatric glioblastoma, IDH wildtype, subclass RTK2a (GBM_pedRTK2a); pediatric glioblastoma, IDH wildtype, subclass RTK2b (GBM_pedRTK2b).

**Supplementary Material 3: Figure S3. Histopathological and molecular features of the case #11.** The case #11 presented classical features of HGG-MYCN such as a dense proliferation composed of large cells with prominent nucleoli (HPS, magnification x400) with overexpression of p53 (magnification x400). The FISH analysis failed to reveal any amplification of *MYCN* and *ID2* loci, but there was an amplification of *MYC* gene (green signals, orange signals: centromere of chromosome 8) (magnification x800). There was no germline mutation of *TP53*. FISH: Fluorescence in situ hybridization; HGG: high-grade glioma; HPS: Hematoxylin Phloxin Saffron; mut.: mutation. Black scale bars represent 50 μm.

**Supplementary Material 4:** Detailed clinical, histopathological, genetic and epigenetic characteristics of the cohort.

## Data Availability

Not applicable.
