# Supplementary figures and images for: Refinement of diagnostic criteria for pediatric-type diffuse high-grade glioma, IDH- and H3-wildtype, MYCN-subtype including histopathology, TP53, MYCN and ID2 status
Source: Acta Neuropathol Commun. 2023 Oct 24;11:170. doi: 10.1186/s40478-023-01667-x (PMC10594904; doi:10.1186/s40478-023-01667-x)

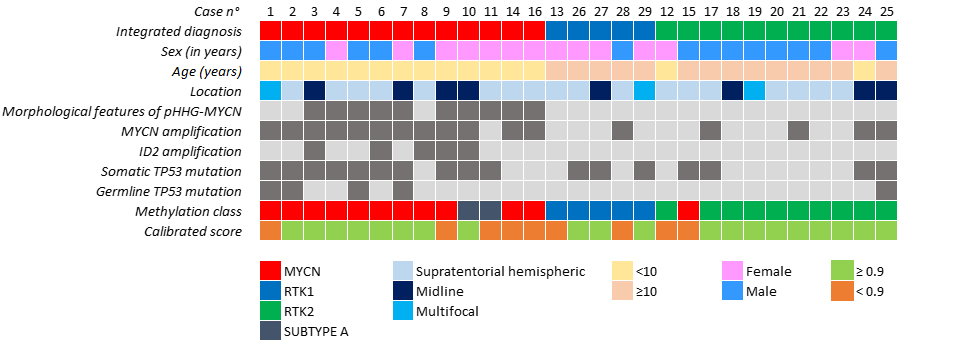

Supplement: Supplementary file 1 — Supplementary Material 1: Figure S1. Clinical, histopathological, genetic and epigenetic characteristics of the cohort. [file 40478_2023_1667_MOESM1_ESM.tif]

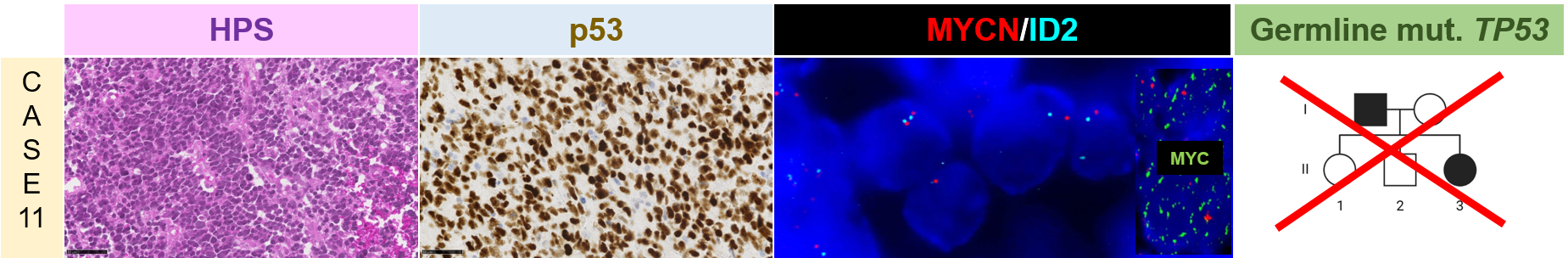

Supplement: Supplementary file 2 — Supplementary Material 2: Figure S2. t-distributed stochastic neighbor embedding (t-SNE) analysis of DNA methylation profiles of the investigated tumors alongside selected reference samples. Reference DNA methylation classes: diffuse midline glioma H3 K27M mutant (DMG_K27); diffuse midline glioma EGFR-altered (DMG_EGFR); diffuse high-grade glioma, H3.3 G34 mutant (GBM_ G34); pediatric glioblastoma, IDH wildtype, subclass MYCN (GBM_pedMYCN); pediatric glioblastoma, IDH wildtype, subclass RTK1a (GBM_pedRTK1a); pediatric glioblastoma, IDH wildtype, subclass RTK1b (GBM_pedRTK1b); pediatric glioblastoma, IDH wildtype, subclass RTK1c (GBM_pedRTK1c); pediatric glioblastoma, IDH wildtype, subclass RTK2a (GBM_pedRTK2a); pediatric glioblastoma, IDH wildtype, subclass RTK2b (GBM_pedRTK2b). [file 40478_2023_1667_MOESM2_ESM.tif]

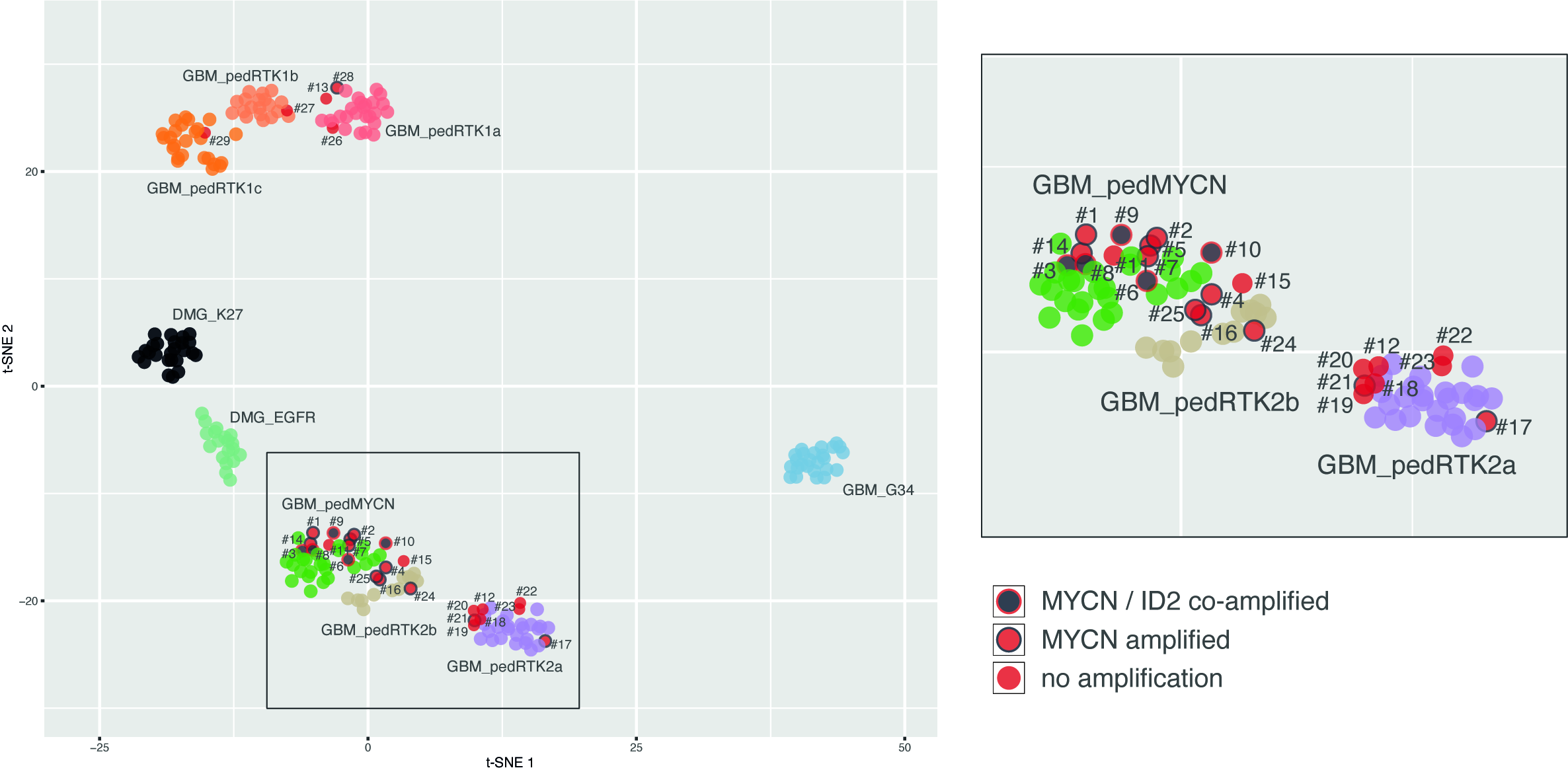

Supplement: Supplementary file 3 — Supplementary Material 3: Figure S3. Histopathological and molecular features of the case #11. The case #11 presented classical features of HGG-MYCN such as a dense proliferation composed of large cells with prominent nucleoli (HPS, magnification x400) with overexpression of p53 (magnification x400). The FISH analysis failed to reveal any amplification of MYCN and ID2 loci, but there was an amplification of MYC gene (green signals, orange signals: centromere of chromosome 8) (magnification x800). There was no germline mutation of TP53. FISH: Fluorescence in situ hybridization; HGG: high-grade glioma; HPS: Hematoxylin Phloxin Saffron; mut.: mutation. Black scale bars represent 50 μm. [file 40478_2023_1667_MOESM3_ESM.tif]
